# Cell Wall Ingrowths in Nematode Induced Syncytia Require *UGD2* and *UGD3*

**DOI:** 10.1371/journal.pone.0041515

**Published:** 2012-07-26

**Authors:** Shahid Siddique, Miroslaw Sobczak, Raimund Tenhaken, Florian M. W. Grundler, Holger Bohlmann

**Affiliations:** 1 Division of Plant Protection, Department of Crop Sciences, University of Natural Resources and Life Sciences, Vienna, Austria; 2 Department of Botany, Warsaw University of Life Sciences (SGGW), Warsaw, Poland; 3 Division of Plant Physiology, University of Salzburg, Salzburg, Austria; Iowa State University, United States of America

## Abstract

The cyst nematode *Heterodera schachtii* infects roots of Arabidopsis plants and establishes feeding sites called syncytia, which are the only nutrient source for nematodes. Development of syncytia is accompanied by changes in cell wall structures including the development of cell wall ingrowths. UDP-glucuronic acid is a precursor of several cell wall polysaccharides and can be produced by UDP-glucose dehydrogenase through oxidation of UDP-glucose. Four genes in Arabidopsis encode this enzyme. Promoter::GUS analysis revealed that *UGD2* and *UGD3* were expressed in syncytia as early as 1 dpi while expression of *UGD1* and *UGD4* could only be detected starting at 2 dpi. Infection assays showed no differences between Δ*ugd1* and Δ*ugd4* single mutants and wild type plants concerning numbers of males and females and the size of syncytia and cysts. On single mutants of Δ*ugd2* and Δ*ugd3*, however, less and smaller females, and smaller syncytia formed compared to wild type plants. The double mutant ΔΔ*ugd23* had a stronger effect than the single mutants. These data indicate that *UGD2* and *UGD3* but not *UGD1* and *UGD4* are important for syncytium development. We therefore studied the ultrastructure of syncytia in the ΔΔ*ugd23* double mutant. Syncytia contained an electron translucent cytoplasm with degenerated cellular organelles and numerous small vacuoles instead of the dense cytoplasm as in syncytia developing in wild type roots. Typical cell wall ingrowths were missing in the ΔΔ*ugd23* double mutant. Therefore we conclude that *UGD2* and *UGD3* are needed for the production of cell wall ingrowths in syncytia and that their lack leads to a reduced host suitability for *H. schachtii* resulting in smaller syncytia, lower number of developing nematodes, and smaller females.

## Introduction

According to their lifestyle, root parasitic nematodes can be divided into sedentary and migratory parasites. Sedentary nematodes induce sophisticated feeding sites in the roots and these feeding sites are the sole source of nutrients throughout their life. There are two important groups of sedentary endoparasitic nematodes: the cyst nematodes, which include the genera *Heterodera* and *Globodera*; and the root-knot nematodes, which include the genus *Meloidogyne*. Cyst and root-knot nematode infection results in the formation of feeding sites termed “syncytia” and “giant cells”, respectively. Cyst nematodes are of agricultural concern mainly in soybean (*H. glycines*), sugarbeet (*H. schachtii*), wheat (*H. avenae*) and potato (*G. pallida* and *G. rostochiensis*).

*H. schachtii* is a major parasite of sugarbeet but also infects brassicaceous plants such as cabbage, oil seed rape, and *Arabidopsis thaliana*. The interaction between *H. schachtii* and Arabidopsis has been developed into a well established model system [1]. Infective second stage juveniles (J2) invade the plant roots near the root tip and migrate intracellularly towards the vascular cylinder where they select a single cell (initial syncytial cell, ISC) to induce a syncytium. After an ISC is selected, the nematode remains motionless for 6–8 h and prepares for feeding in accordance with its sedentary mode of life (feeding preparation period) [2]. Once the feeding preparation period is completed the nematode starts feeding and the ISC develops into a syncytium by incorporation of neighbouring cells [3].

During the following two weeks, nematodes continue to draw nutrients from syncytia and develop into males or females after molting three times (J3, J4 and adult). A female-associated syncytium is composed of around two hundred cells [4] and reaches its maximum size about 10 days after infection [5]. Cyst nematodes are dimorphic but the mechanism of sex determination is not clearly understood. Under favourable conditions with a sufficient supply of nutrients, the majority of juveniles develop into females. However, when juveniles are exposed to adverse growth conditions, e.g. in resistant plants, the percentage of male nematodes increases considerably [6]. Factors such as penetration rate, size of nematodes, size and development of syncytium, and number of eggs are all influenced by the host plant [7], [8], [9].

The root cells that are incorporated into the syncytium undergo drastic ultrastructural changes. Their nuclei become hypertrophied and the cytoplasm condenses with increasing numbers of mitochondria, plastids, ribosomes and structures of endoplasmic reticulum. Additionally, the large central vacuole is replaced by several small vacuoles [10]. All these structural modifications within the syncytium require massive transcriptomic and metabolomic changes within the host cells that have been studied extensively [9], [11], [12], [13], [14]. An important aspect of this reorganization inside syncytia is the modification of cell walls [9], [11], [12]. On one hand, cell walls between the syncytial elements are locally dissolved to incorporate neighbouring cells into the syncytium [10], [15]. On the other hand, outer cell walls are extended and thickened to withstand the increased turgor pressure inside the syncytium [10], [12]. The first change in cell wall structure appears just after the selection of an ISC. A thin layer of electron translucent material is deposited on the walls of the ISC and a few neighbouring cells [16]. Currently, not much is known about the composition of syncytial cell walls. However, some histological studies have shown the presence of cellulose, hemicelluloses and pectins [16], [17]. In addition to these changes, syncytia associated with female nematodes develop pronounced cell wall ingrowths at the interface with xylem vessels, similar to the ones found in transfer cells of plants [16], [18], [19], [20]. These ingrowths are first observed at the end of the J3 stage and show the same staining pattern as observed for the remaining syncytial cell walls. The plasmalemma of the syncytium follows the outline of cell wall ingrowths and many cellular organelles are situated in the vicinity of these ingrowths. In J4 and adult females, earlier formed cell wall ingrowths are fused to form distinct depositions on cell walls of the syncytium. However, new ingrowths are continuously formed [10], [16], [21], [22]. The occurrence of cell wall ingrowths leads to an increase in surface area of the plasma membrane at the interface between syncytium and xylem elements, which in turn increases short distance water and nutrient transport into the syncytium. In contrast to females, cell wall ingrowths in syncytia associated with a male nematode are short and stubby [22]. Additionally, these are immediately fused after deposition. The described cell wall modifications require the activity of cell wall modifying enzymes. Indeed, expression of cell wall modifying proteins such as expansins, endoglucanases, methyl esterases and extensins has been detected in syncytia as well as in surrounding tissues [for review see 21]. On the other hand, cell wall polysaccharides have to be synthesized for the thickening of the outer cell wall of the syncytium and the production of cell wall depositions.

**Figure 1:**
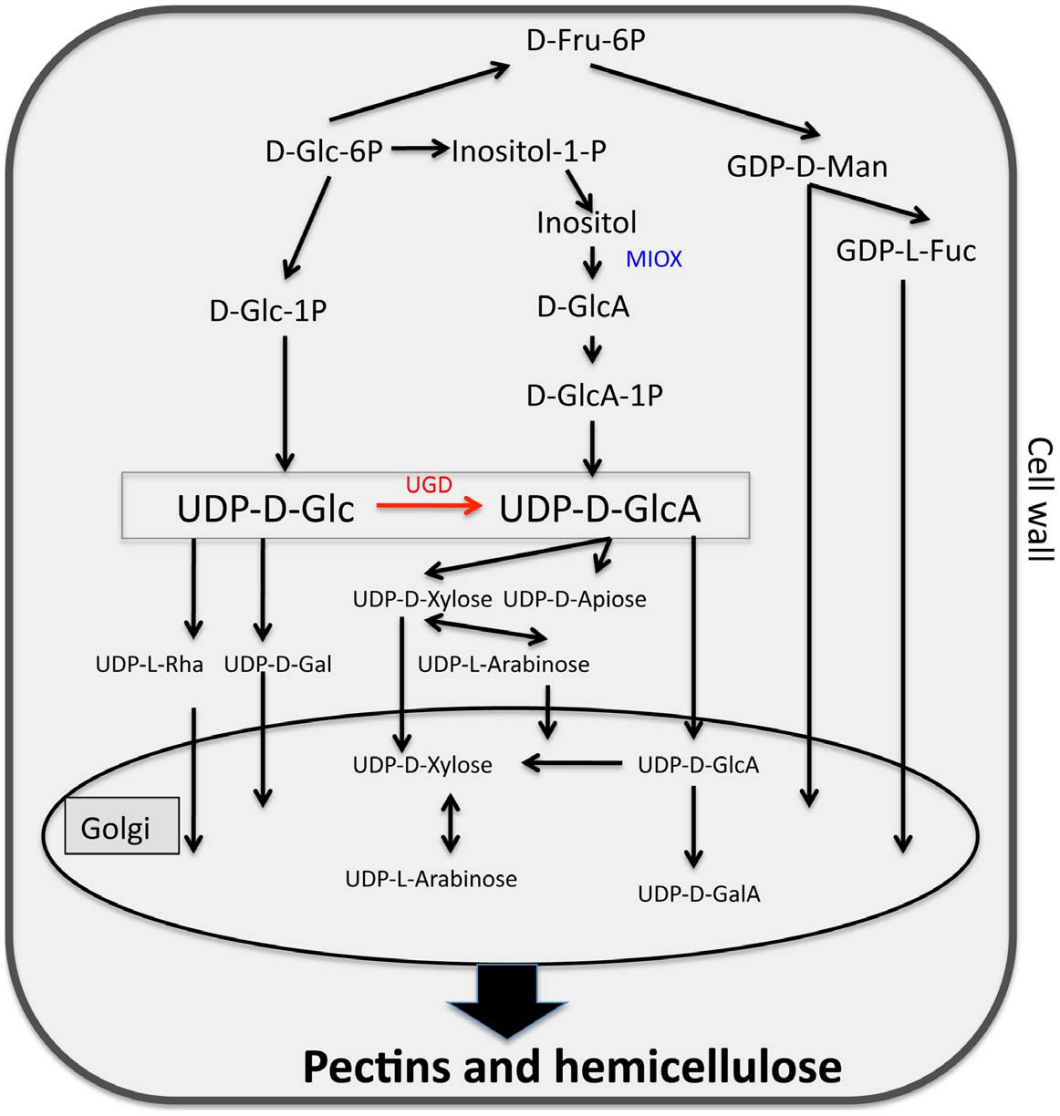
Pathways for the synthesis of UDP-Glucuronic acid and derivatives as adapted from [**24**]. UDP-D-GlcA is a precursor of several cell wall polysaccharides and is synthesized by two pathways. The main pathway involves oxidation of UDP-D-Glc into UDP-D-GlcA by enzyme UGD (Red). Alternative involves conversion of inositol into D-GlcA by enzyme MIOX (blue). D-GlcA is further converted into UDP-D-GlcA. UDP-D-GlcA: UDP-D-glucuronic acid, UDP-D-Glc: UDP-glucose, UGD: UDP-glucose dehydrogenase, MIOX: myo-inositol oxygenase, UDP-D-GalA: UDP-D-galacturonic acid, UDP-Gal: UDP-D-galactose, UDP-Rha: UDP-L-rhamnose, D-Glc-6P: Glucose-6-phospahate, GDP-D-Man: GDP-D-mannose, GDP-L-Fuc: GDP-L-fucose, D-Fru-6P: D-Fructose-6-phospahate, Inositol-1-P: Inositol-1-phosphate, D-Glc-1P: D-Glucose-1-phospahate, D-GlcA: D-glucuronic acid.

UDP-glucuronic acid (UDP-GlcA) is a major precursor for several cell wall polysaccharides. Two pathways contribute to the synthesis of this nucleotide sugar in plants (Figure 1). Under normal growth conditions, the pathway via the enzyme UDP-glucose dehydrogenase (UGD) is dominant for UDP-GlcA production [23], [24]. UGD oxidizes UDP-glucose to UDP-GlcA. A family of 4 genes in the Arabidopsis genome encodes UGD (*UGD1*, *UGD2*, *UGD3* and *UGD4*). *UGD1* has a weak expression in roots while the other 3 genes (*UGD2*, *UGD3* and *UGD4*) are strongly expressed in roots [25]. *UGD1* has generally a low expression level and *UGD2* and *UGD3* show a very strong expression in stems. According to GeneChip data published by Szakasits et al. [14], all four *UGD* genes are expressed in syncytia induced by *H. schachtii* in Arabidopsis roots at 5 and 15 dpi. However, *UGD2* and *UGD3* did not show a difference in expression level in syncytia compared to control root segments. Expression of *UGD1* was significantly upregulated in syncytia compared to control root segments while expression of *UGD4* was significantly down-regulated.

The myo-inositol oxygenase (MIOX) pathway produces additional UDP-GlcA from myo-inositol. The key enzyme of this pathway is myo-inositol oxygenase (MIOX), which is encoded by a family of 4 genes in the Arabidopsis genome. *MIOX1* and *MIOX2* are expressed in seedlings while *MIOX4* and *MIOX5* are highly expressed in pollen [26]. During a previous study, we observed that all 4 *MIOX* genes were expressed at high levels in syncytia, indicating the importance of this pathway for the development of syncytia. Indeed, double mutants of the 4 *MIOX* genes showed significantly reduced development of *H. schachtii* [9]. However, we did not find evidence that the lower susceptibility of *MIOX* double mutants might be due to an impairment in cell wall synthesis as could have been concluded since it is known that the MIOX pathway leads to the production of the cell wall polysaccharide precursor UDP-GlcA. Therefore, the significance of this pathway for the production of UDP-GlcA in plants and syncytia remained unknown. Recently, Endres et al. [27] reported a quadruple mutant for all *MIOX* genes in Arabidopsis, but also in this case no change in cell wall structure and composition was detected [27].

In the current study we investigated the role of *UGD* genes for the development of syncytia and nematodes on Arabidopsis roots. We report here the expression analysis of *UGD* genes in Arabidopsis roots infected with *H. schachtii*. The analysis of the *UGD* mutants showed that *UGD2* and *UGD3* but not *UGD1* and *UGD4* are important for the development of syncytia and nematodes. Our results indicate that *UGD2* and *UGD3* are essential for the formation of cell wall ingrowths on the outer cell walls of syncytia.

## Results

### *UGD*expression Analysis

**Figure 2:**
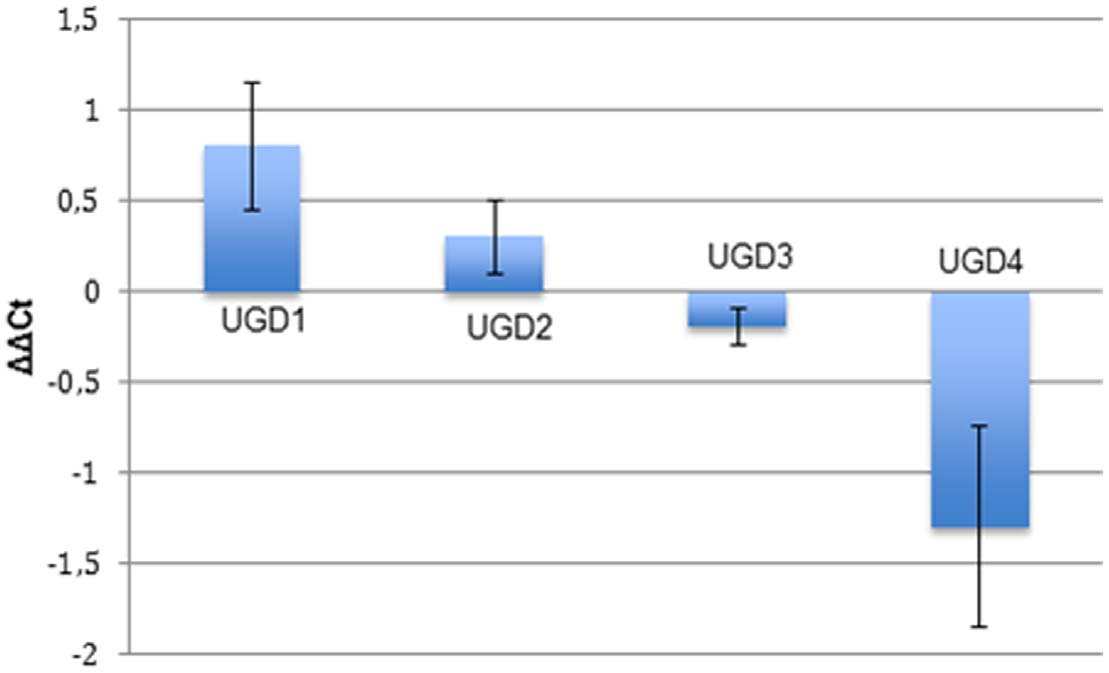
Expression of *UGD* genes in syncytia. Expression of *UGD* genes as measured by qRT-PCR relative to the expression of 18 S RNA. Transcripts were measured from root segments containing syncytium at 15 dpi and compared with corresponding control root segments.

A recent transcriptome analysis [14] had shown that expression of *UGD1* was significantly upregulated in syncytia compared to control root segments (Table S1) while the expression of *UGD2* and *UGD3* remained unchanged. Expression of *UGD4* was significantly downregulated in syncytia compared to control roots. We confirmed these data using qRT-PCR of syncytia that were cut out from infected roots (Figure 2). The results obtained for the four *UGD* genes were in line with the GeneChip data.

**Figure 3:**
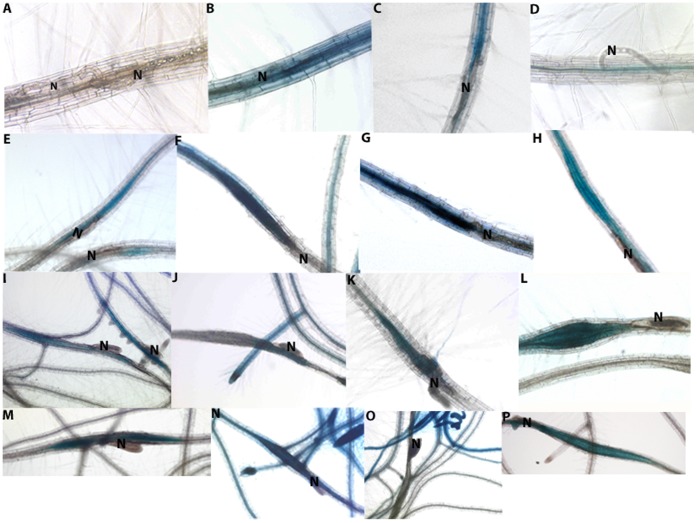
Expression of GUS driven by *UGD* promoters in infected Arabidopsis roots. At 1 dpi GUS expression was observed in *UGD2*, *UGD3* and *UGD4* lines. No expression was observed in the *UGD1* line. At 2 dpi and 7 dpi expression of all four *UGD* genes was detected in syncytia. In addition, *UGD2* and *UGD3* expression was also observed in tissues outside syncytia. Expression of *UGD4* sometimes extended beyond the syncytium (*UGD4,* 7 dpi). Expression of all *UGD* genes was detected in 10 dpi syncytia. Promoter::*GUS* assay of *UGD1* (A, E, I and M), *UGD2* (B, F, J and N), *UGD3* (C, G, K and O) and *UGD4* (D, H, L and P) at 1 dpi (A–D), 2 dpi (E–H), 7 dpi (I–L) and 10 dpi (M–P). N-nematode.

For a more detailed analysis of *UGD* gene expression in syncytia we performed GUS expression analysis of promoter::GUS fusions for all four *UGD* genes was carried out in a time course analysis starting as early as 1 dpi. We did not see any GUS expression for *UGD1* at 1 dpi (Figure 3A). At this time point, GUS staining was observed for *UGD2* and *UGD3* in infected tissues (Figure 3B and 3C). For *UGD4*, expression was present only in a few syncytia and spread also into the vicinity of syncytia (Figure 3D). At 2 dpi, GUS expression was present in syncytia for all four *UGD* genes (Figure 3E–2H). However, reporter activity was strongest in case of *UGD2* and *UGD3*. The majority of syncytia showed a moderate GUS expression for *UGD1* at 2 dpi. However, a significant (approximately 25%) number of syncytia did not show any specific staining for *UGD1* at 2 dpi. At 3 dpi, all four *UGD* genes showed the same expression pattern as at 2 dpi (data not shown). At 7 dpi, expression of all four *UGD* genes was detected in syncytia (Figure 3I–2N). This expression was most intense in the case of *UGD2* and *UGD3*, similar to the pattern observed at 2 dpi. At 10 dpi, all four genes were expressed in syncytia associated with female nematodes. However, in the case of *UGD3* expression was turned off in the majority (approximately 63%) of syncytia (Figure 3O). There was no expression detected for any of the *UGD* genes in syncytia at 10 dpi associated with male nematodes (Figure S1). In general, *UGD2* and *UGD3* showed a similar expression pattern throughout the analysis. However, in addition to syncytia, both of these genes were also expressed in uninfected parts of the roots. *UGD1* showed occasional expression within the vascular cylinder (Figure S2A and S2B). *UGD2* was expressed in lateral roots and in the elongation zone of the roots (Figure S2C and S2D). *UGD3* was strongly expressed in young parts of the lateral roots whereas its expression was almost absent in older roots (Figure 3K, 3O and S2E). For *UGD4*, a rather weak GUS expression was found in control roots but a strong expression was occasionally observed in lateral roots especially in the elongation zone of young lateral roots (Figure S2F). This weak GUS expression of *UGD4* is in contrast to gene expression data from AtGenexpress, which are based on a GeneChip analysis of the root transcriptome [25]. In addition to AtGenexpress, we also extracted the expression data of the four *UGD* genes from the AREX database (Figure S3).

### Infection Assays

**Figure 4:**
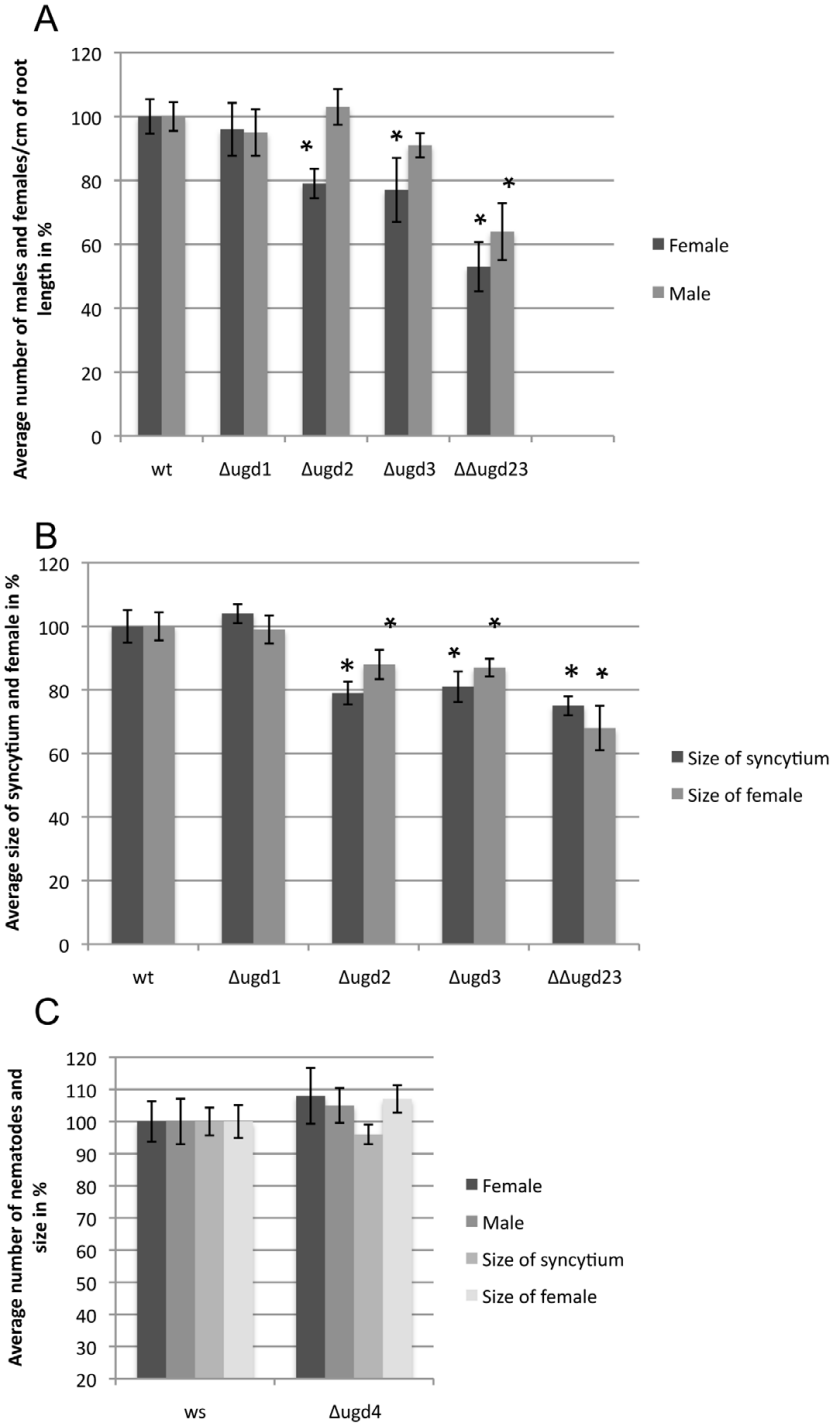
Infection assay of single and double knockout mutants for *UGD* genes. Average numbers of males and females were counted at 14 dpi and were normalized to the length??/area of the root. The wild type was set as 100%. Size of syncytia and associated females were measured at 14 dpi. 20–30 syncytia and attached females were randomly selected and their sizes were determined. These values were converted into percentages (%) setting the wild type values as 100%. Three independent replicates were carried out. Values are means ±SE (standard error). Data were analysed for significant difference using ANOVA (p<0.05) and LSD tests. Asterisks indicate significant difference to wild type. (A) Numbers of males and females for Col wild type, Δ*ugd1*, Δ*ugd2*, Δ*ugd3,* and ΔΔ*ugd23* mutants. (B) Sizes of females and associated syncytia for Col wild type, Δ*ugd1*, Δ*ugd2*, Δ*ugd3,* and ΔΔ*ugd23* mutants at 14 dpi. (C) No *UGD4* mutant in the Columbia background was available and therefore a mutant in the WS background was used. Number of males and females were counted for Δ*ugd4* at 14 dpi and were normalized to the length??/area of root. Similarly, areas of syncytia and females were counted at 14 dpi.

We have previously shown the importance of *MIOX* genes for the development of syncytia. Since all *UGD* genes were expressed in syncytia and since UGD is also involved in the synthesis of UDP-GlcA, we were interested to test if *UGD* genes are also needed for the development of syncytia. We first tested the available *UGD* single T-DNA knockout mutants. The mutants Δ*ugd1,* Δ*ugd2*, and Δ*ugd3* were in the Col background and were compared with the Col wild type (Figure 4A and B) while Δ*ugd4* was in the Ws background and was therefore compared to the Ws wild type (Figure 4C). Two weeks after inoculation, when males and females could be clearly distinguished, the numbers of females and males were counted. No significant difference was observed in case of Δ*ugd1* and Δ*ugd4* single mutants. However, Δ*ugd2* and Δ*ugd3* showed a significant reduction (20%) in the average number of females compared to wild type plants (Figure 4A). There was no change in the average number of males in these lines. In addition to the average numbers of nematodes, we also measured the size of syncytia and the size of females (Figure 4B and 4C). We observed that Δ*ugd2* and Δ*ugd3* but not Δ*ugd1* and Δ*ugd4* resulted in a significant reduction in the size of syncytia and females. Thus, only *UGD2* and *UGD3* but not *UGD1* and *UGD4* had a significant effect on syncytium and nematode development. We therefore also used the available double mutant ΔΔ*ugd23* in a nematode infection assay as described above. This double mutant showed different phenotypes when grown on MS agar [24]. Some seedlings had no primary root but developed secondary roots several days after germination. Other seedlings had shorter primary roots and the secondary roots developed one or two days later than the wild type, but otherwise they looked normal. It is important to note that we used only those plants which developed primary roots for analysis and that we corrected the data for the decreased size of the ΔΔ*ugd23* double mutant by referring to average root length (see details in Material and Methods). Compared to the single mutants, the number of female nematodes on the double mutant was drastically reduced to only 50–60% of the wild type level. In addition, the ΔΔ*ugd23* double mutant also showed a 40% reduction in the number of male nematodes. Although not significant, the number of males that developed on the ΔΔ*ugd23* double mutant seemed to be higher than the number of females. The size of females and syncytia was reduced by 30%.

### Ultrastructural Analysis of Syncytia

**Figure 5:**
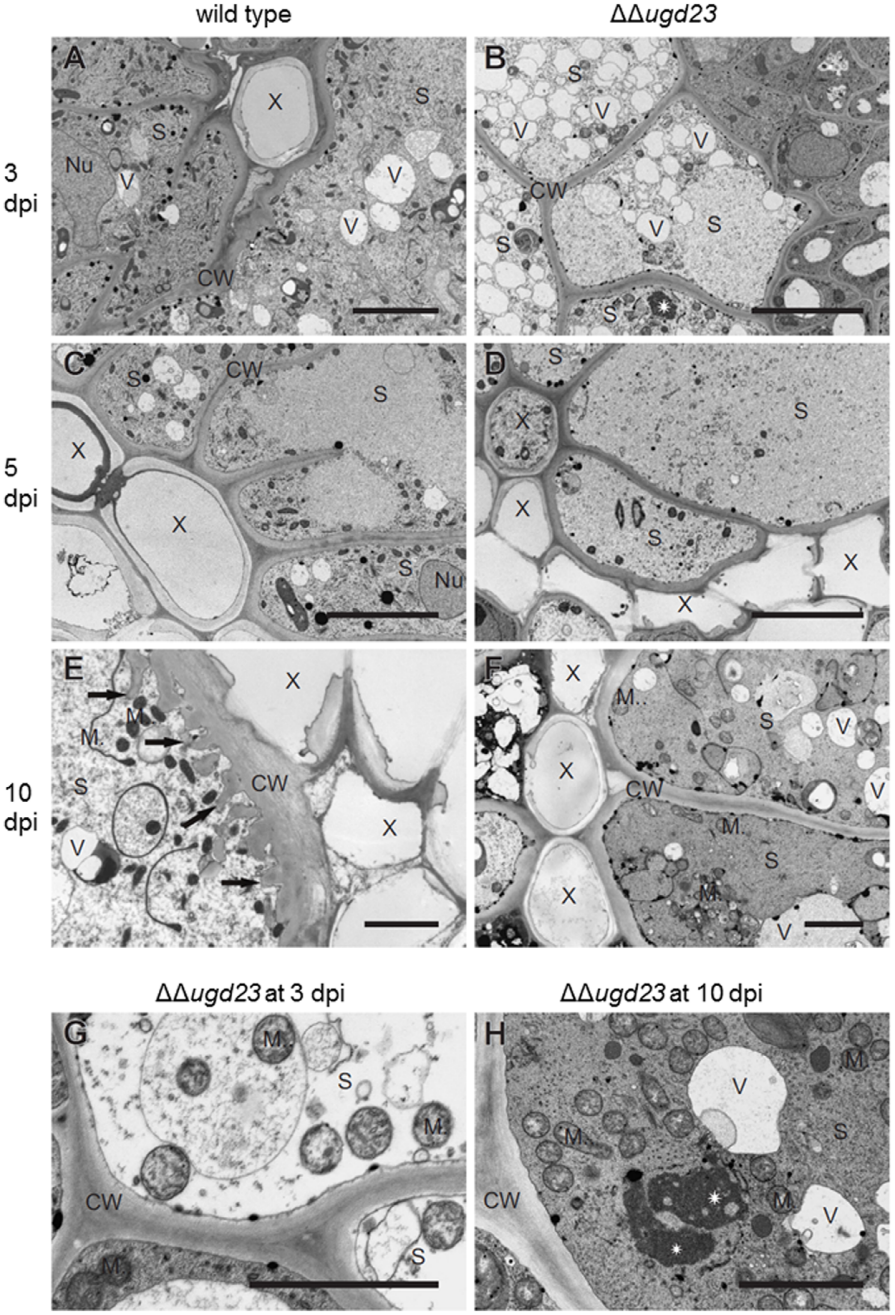
Electron microscope comparison of syncytia developed in wild type and ΔΔ***ugd23*** **mutant roots.** Syncytia induced in ΔΔ*ugd23* mutant roots at 3 dpi lacked electron dense cytoplasm and numerous organelles are degenerated compared to those induced in wild-type roots. 5 dpi syncytia in ΔΔ*ugd23* mutant revealed a similar ultrastructure as observed in 3 dpi syncytia. 10 dpi syncytia associated with a female nematode in roots of ΔΔ*ugd23* mutants lacked cell wall ingrowths compared to wild type syncytia. Sections of syncytia induced in wild type (A, C, and E) and ΔΔ*ugd23* (B, D, and F–H) plants collected at 3 (A, B and G), 5 (C and D) and 10 (E, F, and H) dpi. Scale bars 5 (A–D) and 2 (E–H) µm. CW: cell wall, M: mitochondrion, N: nucleus, S: syncytium, X: xylem, V: vacuole, arrows: cell wall ingrowths (E). Asterisk: chromatin/nucleolus clumps (B and H).

The strong effect of the double mutant prompted us to study the development of syncytia at the ultrastructural level in a time course analysis at 3 dpi, 5 dpi and 10 dpi (Figure 5). We also compared the anatomy of uninfected roots of the ΔΔ*ugd23* double mutant and of wild type plants, but neither ultrastructural nor anatomical differences could be found (Figure S4F and S4G versus S4A and S4B). However, investigations on syncytia revealed significant changes in roots of the ΔΔ*ugd23* mutant. In general, syncytia induced in roots of ΔΔ*ugd23* mutant were smaller than those induced in wild type plants (Figure S4H–J versus S4C–E). Additionally, indications of aberrant syncytium development similar to senescence were observed at all time points studied. At 3 dpi, syncytia developing in wild type plants contained an electron dense cytoplasm with numerous organelles (Figure 5A). Dense cytoplasm was lacking in syncytia from ΔΔ*ugd23* mutant roots (Figure 5B). Instead, they contained an electron translucent cytoplasm with numerous small vacuoles. Cellular organelles, especially mitochondria and structures of endoplasmic reticulum, looked changed or degenerated (Figure 5F–H). The mitochondria had an electron translucent matrix and short and dilated cristae (Figure 5G) even in those few syncytia that still had electron dense cytoplasm (Figure 5H). At all examined developmental time points of syncytia clumps of chromatin or nucleoli remnants without membrane envelope were present in syncytia of the the ΔΔ*ugd23* mutant (Figure 5B and H). A similar situation was observed in syncytia at 5 dpi (Figure 5C and D). Again, most of the examined syncytia developing in ΔΔ*ugd23* roots contained an electron translucent cytoplasm with aberrant organelles and nuclei (Figure 5D). Only some of them contained dense cytoplasm albeit with degenerated organelles. Short and collapsed cell wall ingrowths were observed in only one of the syncytial samples at 5 dpi and cell wall ingrowths were missing in all samples collected from wild type plants at the same time point (Figure 5C). At 10 dpi, syncytia induced in ΔΔ*ugd23* roots appeared degenerated, containing many vacuoles and aberrant organelles. Cell wall protuberances were not found in these samples (Figure 5F) compared to wild-type syncytia where these were present in all samples examined (Figure 5E). In some cases syncytia in the double mutant developed normally with an electron dense cytoplasm, similar to that found in syncytia induced in roots of wild-type Arabidopsis plants. Also in these syncytia we did not find cell wall ingrowths (data not shown).

### Cell Wall Composition Analysis

**Figure 6:**
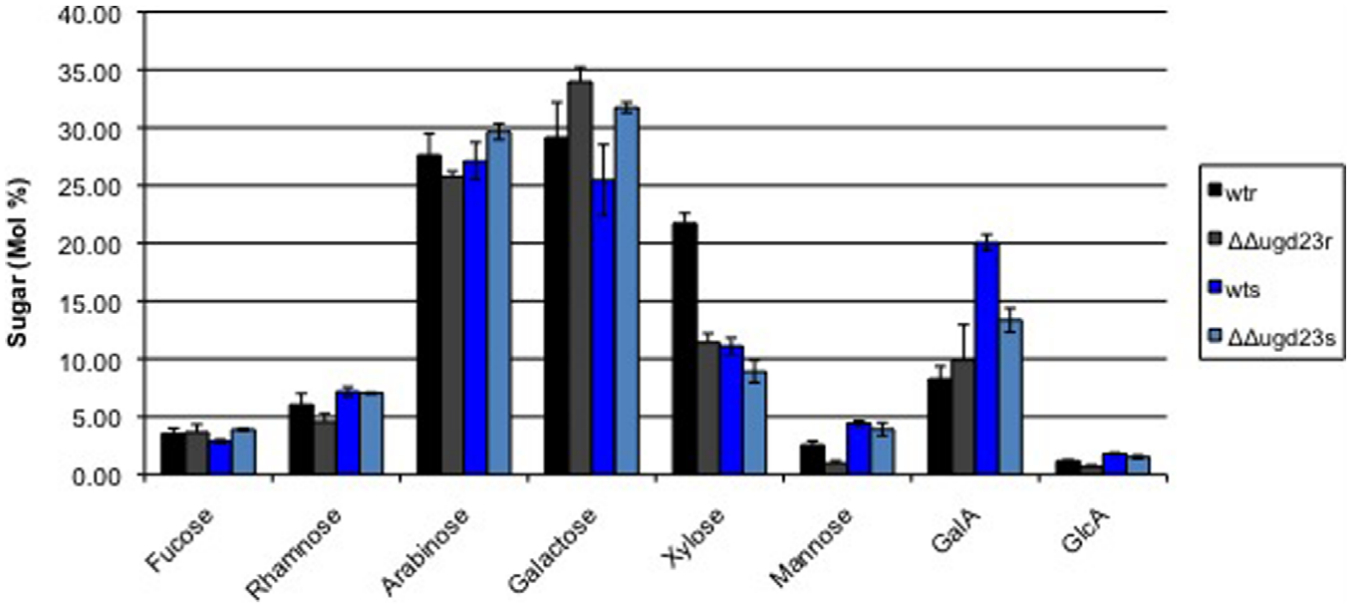
Cell wall sugars in syncytia and roots of wild type plants and ΔΔ*ugd23* mutant at 14 dpi. The sugar composition of cell walls was analysed via HPLC anion-exchange column. Values are mol% ±SE. Each measurement was carried out in at least three independent replicates. wtr: wild type root, ugd23r: *ugd23* root, wts: wild type syncytium, ugd23s: *ugd23* syncytium.

The drastic changes in ultrastructure of syncytia induced in the roots of the ΔΔ*ugd23* double mutant as well as a recent study of cell walls in leaves of the ΔΔ*ugd2*3 double mutant [24] indicated a possible change in cell wall composition of syncytia. We therefore compared the cell wall composition of syncytia in wild type roots with that of syncytia in ΔΔ*ugd23* double mutant roots. Syncytia associated with female nematodes were dissected (after removing the nematodes) from wild type as well as ΔΔ*ugd23* double mutant roots at 15 dpi. These samples as well as segments from control roots were analysed by high performance anion exchange chromatography and electrochemical pulsed amperometric detection (HPAEC-PAD) for differences in the amount of cell wall monomer sugars (Figure 6). Our analysis showed that uninfected roots of the ΔΔ*ugd23* mutant contained significantly less xylose (−43%) mannose (−58%) and glucuronic acid (−37%) compared to uninfected roots of wild type plants. Similarly, root segments of the double mutant ΔΔ*ugd23* harboring syncytia contained significantly less xylose (−20%), galacturonic acid (−33%), and glucuronic acid (−16%) but more galactose (+25%) compared to corresponding segments from wild type Arabidopsis plants.

### Immunolabelling of Cell Wall Polymers in Roots and Syncytia

Since cell wall composition cannot be determined for pure syncytium material, we also used immunolabelling as an additional method allowing direct examination of syncytial cell walls. Monoclonal antibodies recognizing particular cell wall epitopes were used to test for larger quantitative differences of certain polymers in the cell wall of syncytia from wild type and ΔΔ*ugd23* roots. We focused on three antibodies: LM5 (galactan), LM6 (arabinan) and JIM7 (partially methylated pectin) because these antibodies had revealed differences in hypocotyl cell walls of wild type and ΔΔ*ugd23* double mutant plants before [24]. However, as shown in supplementary Figure S5, the cell walls of the syncytia did not show major differences in the labelling pattern and signal strength when we compared syncytia from wild type and ΔΔ*ugd23* roots.

## Discussion

Cell wall ingrowths are generally found in transfer cells which are ubiquitously distributed in all plants [19]. Ingrowths lead to an increase of the area at the boundary between symplast and apoplast and thus allow a higher exchange rate of solutes and nutrients. Transfer cells have for instance been studied in the endosperm of cereals where they are involved in the import of nutrients into the embryo and in minor leaf veins where they also facilitate loading and unloading of nutrients. Not much is known to date about genes that are involved in regulating the formation of cell wall ingrowths. Recently, GIGANTEA was identified as a regulator of cell wall ingrowths differentiation in Arabidopsis phloem parenchyma transfer cells of leaf minor veins [28]. In *Vicia faba* cotyledons reactive oxygen species were found to be involved in initiation of cell wall ingrowths [29]. A transcriptome study of barley endosperm transfer cells identified 406 upregulated genes using a 12 K makroarrray [30]. Since the formation of cell wall ingrowths requires cell wall synthesis, it is not surprising that 17 genes coded for cell wall metabolism enzymes. UDP-GlcA is an important precursor for a variety of plant cell wall polysaccharides, contributing to about 50% of cell wall biomass in Arabidopsis [31]. In Arabidopsis UDP-GlcA is mainly produced by UGD [23], [24]. Additionally, the MIOX pathway contributes UDP-GlcA in certain tissues including syncytia [9].

### Different Timing of Expression of *UGD* and *MIOX* Genes in Response to Nematode Infection

In this paper, we studied the expression of all four *UGD* genes in response to infection with the beet cyst nematode *H. schachtii* by using qRT-PCR and promoter::GUS analysis. Expression of *UGD2* and *UGD3* in syncytia was detected throughout the expression analysis starting as early as 1 dpi. However, in addition to syncytia, these genes were also expressed in uninfected roots. In comparison to *UGD2* and *UGD3*, *UGD1* and *UGD4* showed a rather syncytium specific expression pattern, as GUS staining of these promoter::GUS constructs in uninfected roots was not as strong as for *UGD2* and *UGD3*. The results for *UGD4* seemed to be in contrast to other data (Figure S3 and [25]) showing a strong root-specific expression. However, this strong expression was found in young roots, while older uninfected roots (Figure S2) showed a much weaker expression. Expression of all four *UGD::GUS* fusions at 10 dpi was only detected in syncytia associated with female nematodes. At this time point, both sexes can be clearly distinguished and the males will start to leave their feeding site to mate with females. A reason for this difference in promoter activity might be that cell wall ingrowths, which require cell wall synthesis, are formed mostly in syncytia associated with female nematodes as these need to take up more nutrients from the feeding site than the male nematodes to support the development of the eggs. Indeed, it has been shown that female juveniles require 3 times more food compared to a male until moulting to adult stage. Average food consumed during the life of a female was found to be 29 times greater than that of males [32]. We also observed that none of the *UGD::GUS* fusions showed expression within syncytia at 15 dpi. This is in contrast to GeneChip data [14], where transcripts for all four *UGD* genes were detected at 15 dpi and to the qRT-PCR performed here. An explanation for this difference might be that the transcripts for these genes have a long half-life and are therefore also detectable at these later time points. In a previous study, we have shown that expression of 2 *MIOX* genes (*MIOX4* and *MIOX5*) in syncytia, according to promoter::*GUS* lines, was turned on as late as 5 dpi and could still be detected at 20 dpi. GeneChip data showed that *MIOX2, MIOX4,* and *MIOX5* (*MIOX1* was not included on the GeneChip) are all stongly expressed at 5 dpi and 15 dpi. Thus, the expression of *MIOX* genes continues at later stages of syncytium development and is maintained even when expression of *UGD* genes could not be detected with promoter::*GUS* lines. On the other hand, we were unable to detect expression of *MIOX4* and *MIOX5* genes with promoter::*GUS* fusions before 5 dpi [9]. It is possible that there is a co-ordination in expression between *UGD* and *MIOX* genes during development of syncytia. *UGD* genes might be preferentially involved in the synthesis of cell wall polysaccharides during early stages of syncytium development. However, as the syncytium grows, this pathway is downregulated or unable to produce sufficient amounts of UDP-GlcA to meet the increasing demands of the growing syncytium and developing nematode. As a result, the expression of *MIOX* genes might increase, which are therefore more important at later stages of nematode development. A similar regulation of *MIOX* genes has been observed in experiments, in which plants were kept in the dark after a normal night (extended night) [33]. The depletion of soluble sugars seemed to be the driving force for the induction of *MIOX* genes in that study. More recently, it has been shown that there is a compensatory upregulation of *UGD* genes in *MIOX* quadruple mutants [27], [34]. Thus, the quantitatively more important UGD pathway can rescue a potential *miox* knockout phenotype but MIOX cannot fully rescue knockouts in *UGD* genes, probably because the available myo-inositol is limiting [24].

Similar data for *UGD* and *MIOX* gene expression have been reported for syncytia induced by *H. glycines* in soybean roots [35]. Ithal et al. [35] used Laser Capture Microdissection to collect material for a transcriptome analysis of syncytia. According to the Unigene database, there are nine *UGD* genes in soybean, but only one of them is found on the soybean Affymetrix GeneChip (http://www.ncbi.nlm.nih.gov/UniGene/clust.cgi?UGID=126954&TAXID=3847&SEARCH=gma.6350). This gene has similarity to Arabidopsis *UGD2* and is represented by probeset ID GmaAffx.90733.1.A1_at. Examination of online supplemental data showed that this gene is significantly upregulated (+3.17) in syncytia as early at 2 dpi and is turned off during later stages of nematode development i.e. 10 dpi. These authors also showed that one of the *MIOX* genes (Gma.3888.2.S1_at) was significantly upregulated in syncytia at 5 dpi (+7.77) and 10 dpi (+8.17) compared to 2 dpi. These results support our hypothesis of a co-ordination in expression between *MIOX* and *UGD* genes during syncytium development.

### Function of UGD for Syncytium Development

To test the importance of *UGD* genes for nematode development, we carried out infection assays using T-DNA knock out mutants for *UGD1*, *UGD2*, *UGD3* and *UGD4*. A mutant for *UGD4* was not available in the Col background; therefore, we used a T-DNA mutant in the Ws background. Infection of single mutants did not show any change in the number of nematodes for Δ*ugd1* and Δ*ugd4*. Additionally, the size of syncytia and of female nematodes remained unaltered in these mutants. By contrast, Δ*ugd2* and Δ*ugd3* showed a significant reduction in the number of females while the number of males was not significantly changed. Moreover, the sizes of syncytia and females were smaller compared to those from wild-type plants. These results may be surprising since *UGD4::GUS* and especially *UGD1::GUS* showed a more syncytium specific expression. The absolute expression level of *UGD2* and *UGD3* is however higher and thus these two isoforms contribute mostly to the total UGD activity in plants. It is also very likely that the development of syncytia not only depends on gene expression inside the syncytia but also on gene expression in surrounding tissues. As *UGD2* and *UGD3* showed a similar effect and also a similar expression pattern during syncytium as well as plant development [24], we also studied the ΔΔ*ugd23* double mutant. The Δ*ugd2* and Δ*ugd3* mutants as well as the ΔΔ*ugd23* double mutant have recently been described [24]. In summary, the double mutant plants were much smaller with poor root and shoot growth. All sexual organs were present but flowers had difficulties to open their petals and the number of siliques that contained seeds was reduced significantly. In addition, the double mutant seedlings when grown on MS medium showed three different phenotypes with up to 20% of seedling developing no primary root. However, in this study we used only those plants which developed primary roots for analysis in the nematode infection assay. These plants remain smaller compared to wild type and usually grow to maturity but produce only few seeds [24]. In our analysis we corrected the infection data for the decreased size of the ΔΔ*ugd23* double mutant plants by estimating the length of the roots and all data for the number of nematodes in Figure 4A are per cm root length. These ΔΔ*ugd23* plants showed a reduced support of nematode development. Not only were the numbers of nematodes reduced but the development of nematodes was also affected as revealed by smaller syncytia and smaller female nematodes. The number of male nematodes on the single Δ*ugd2* and Δ*ugd3* mutants was significantly higher than the number of female nematodes. Also the ΔΔ*ugd23* double mutant seemed to support more males than females, although the difference was not significant. A higher number of males indicates that nutrient supply through the syncytia is limited.

Why might UGD be so important for syncytium and nematode development? In Arabidopsis, the major central intermediate for precursors of cell wall polymers is UDP-GlcA based on a polymer analysis of cell walls [36]. Assuming that the UGD pathway is the dominant pathway for production of UDP-GlcA during the early stages of syncytium development, the loss of 2 *UGD* genes will probably reduce the amount of available UDP-GlcA synthesized and hence lead to disturbed cell wall synthesis in syncytia at these early stages. It is therefore not surprising that the ΔΔ*ugd23* mutant showed a reduction of xylose, GalA and GlcA in cell walls of syncytia compared to wild type. However, the amount of galactose was increased significantly in the double mutant syncytia compared to wild type syncytia. This increase in galactose might be due to an increase in the availability of UDP-Glc/UDP-Gal or a compensation for the loss of other cell wall sugars. Leaves from ΔΔ*ugd23* mutant plants have a ∼25% reduction in arabinose, xylose, GalA and >50% reduction in apiose [24]. Taken together, these changes lead to a major change in the pectic polymers, whereas xyloglucan is only slightly less substituted with xylose [24]. The observed changes in the cell wall composition of roots with syncytia suggest similar modifications in these cell walls though not every aspect could be studied with the limited amount of cell wall material from syncytium samples. Furthermore, it has to be taken into account that it is technically impossible to obtain pure cell wall material from syncytia. The syncytium samples that we used also contained material from the surrounding tissues. We therefore also used immunostaining to test for differences in the polysaccharide composition of cell walls from syncytia in wild type and ΔΔ*ugd23* roots. However, no major differences could be detected, which might be explained by the fact that this method can only detect rather large quantitative differences.

It has been shown during previous studies that the sex ratio of juveniles is influenced by the composition of medium, age, physiological state, and carbohydrate status of plants [6], [8], [16], [22]. Under favourable environmental conditions, more females are produced and this trend is reversed under unfavourable conditions. However, in the current study, there was a significant reduction both in numbers of females and males, but without change of the sex ratio. Apparently the triggering factor(s) for epigenetic sex determination are not affected by UGDs and their role in syncytium development.

### UGD is Needed for Cell Wall Ingrowths Formation

We carried out an electron microscopic investigation of syncytium development in ΔΔ*ugd23* double mutants and compared them to those in wild type roots. Our analysis showed that syncytia in ΔΔ*ugd23* mutants lacked the electron dense cytoplasm, which is normally observed in syncytia (Figure5). Aberrant translucent syncytial cytoplasm was observed as early as 3 dpi. Additionally, the majority of syncytia showed early indications of senescence and degradation compared to those that developed on wild type plants. These observations support the view that cell wall biosynthesis is required early for normal development of syncytia. The average size of syncytia and developing nematodes was much smaller in ΔΔ*ugd23* double mutants compared to wild-type plants. Although the majority of syncytia lacked dense cytoplasm, some syncytia contained dense cytoplasm with organelles comparable to that present in wild type syncytia. However, with only one exception, we did not find cell wall ingrowths in the samples from the double mutant. Thus, the lack of cell wall ingrowths in the double mutant was not an indirect effect due to degradation of the cytoplasm. It seems that *UGD2* and *UGD3* but not *UGD1* and *UGD4* are primarily involved in the development of cell wall ingrowths. Cell wall ingrowths are located at the interface to the xylem and are supposed to be important for the water and nutrient supply of syncytia by increasing the surface of the interface area. The absence of cell wall ingrowths may lead to a decrease in uptake into syncytia and subsequently to the other changes that we have observed, such as the absence of the dense cytoplasm and the degradation of mitochondria and nuclei. In consequence, such syncytia are smaller and unable to fully support the development of nematodes, which are therefore reduced in numbers and in size.

### Conclusions

Our studies have shown that all four *UGD* genes are expressed in syncytia induced by the beet cyst nematode *H. schachtii* in Arabidopsis roots. Knocking out *UGD2* and *UGD3* resulted in plants that could only support development of a reduced number of nematodes. Additionally, the sizes of syncytia and female nematodes were decreased significantly in these lines. Ultrastructural and biochemical analysis showed that these genes influenced the normal development of syncytia via cell wall modifications. To further study the function of *UGD* genes in syncytium development, it might be interesting to develop transgenic RNAi lines targeting *UGD* genes driven by syncytium specific promoters. This approach could potentially lead to the development of nematode resistant crop plants. It would also be interesting to perform transcriptomic and metabolomic studies with syncytia from ΔΔ*ugd23* double mutants as this might result in the identification of additional targets for engineering plants with resistance against nematodes.

## Materials and Methods

### Plant Cultivation

*Arabidopsis thaliana* L. wild type and mutant (Δ*ugd1*, Δ*ugd2*, Δ*ugd3*, Δ*ugd4*, and ΔΔ*ugd23*) seeds were surface sterilized for 5 min in 75% ethanol followed by 10 min in 6% (w/v) sodium hypochlorite and subsequently washed three times with sterile water. Seeds were placed into sterile Ø9 cm Petri dishes on a modified Knop medium [1] with 2% sucrose. Seeds were grown in a growth chamber at 25°C in a 16 h light and 8 h dark cycle.

### qRT-PCR

Quantitative real time RT-PCR of *UGD* genes in syncytia and control roots was carried out essentially as described by Siddique et al. [9]. We used the following primers: *UGD1* (5′-AGACTCCAGCGATTGATG-3′; 5′-GAGACGACATTCACTTGC-3′; *UGD2* 5′-AACAACAGTGAAACAAGTGAC-3′; 5′-TGAAACCAATCTCCCTTAGC-3′; UGD3 5′-TGAAGCAAGTCTCAGTCG-3′; 5′-GTAAACAATAAACCCAATCTCC-3′; *UGD4* 5′-TGATGTGTGTAAAGGTCTATTAGG-3′ 5′-ACTGTGGTTGGACTCATTGG-3′. All experiments were repeated three times and all samples were analyzed in three technical replicates.

### GUS Reporter Analysis

The GUS lines have been described before [25]. For analysis of GUS expression, Arabidopsis seeds were surface sterilized and grown on Knop medium as described above. Plants were infected with nematodes as described below (see Nematode infection assay). The plants were analysed for GUS expression in a time course analysis starting at 1 dpi. For GUS expression analysis of control roots, uninfected plants were used. Plant tissues were submerged in 100 mM sodium phosphate buffer (pH 7.0) containing 10 mM EDTA, 0.01% Triton X-100, 0.5 mM K_3_[Fe(CN)_6_], 0.5 mM K_4_[Fe(CN)_6_] and 1 mg ml^−1^ 5-bromo-4-chloro-3-indolyl glucuronide as described in Siddique et al. [9]. Tissues were vacuum infiltrated for 5 min and then incubated in darkness at 37°C for 5–6 h.

### Nematode Infection Assay

*H. schachtii* cysts were harvested from *in vitro* stock cultures maintained on mustard (*Sinapis alba* cv. Albatros) roots growing on Knop medium supplemented with 2% sucrose [1]. Hatching of J2 was stimulated by addition of 3 mM ZnCl_2_. The J2 were resuspended in 0.7% (w/v) gelrite (Duchefa, Haarlem, The Netherlands) and 12 day old Arabidopsis roots were inoculated under sterile conditions with approximately 80–90 J2 per plant. Ten plants were grown in one Petri dish and 3–5 Petri dishes were used for each line. Only seedlings that developed primary roots were analysed. These experiments were repeated three times. Root length was estimated at the date of inoculation as described previously [11], [37]. In short, plants were divided into 5 scoring categories of root length based on development of their roots. The average root length (in cm) of each class has been described previously [11], [37]. Two weeks after inoculation, males and females were counted [9] and were normalized to the length??/area of root. The data were analysed using single factor ANOVA (p<0.05). A Fisher LSD test was applied to further analyse the data.

### Syncytium Size Measurement

In addition to counting the numbers of nematodes, the size of syncytia (associated with females) and of females was also measured as described by Siddique et al. [9]. Briefly, the area of longitudinal optical sections of 14 dpi syncytium (2D) and attached female nematode was measured. For each line, 20–30 syncytia associated with females were randomly selected and photographed using an Axiovert 200 M inverted microscope equipped with a Zeiss Axiocam digital camera (Zeiss, Oberkochen, Germany). The syncytia and females were outlined using the Axiovision Kontour tool and the software calculated the area. These individual measurements were used to calculate the average size of syncytia. Data were further statistically analysed using single factor ANOVA (P<0.05) and LSD tests.

### Electron Microscopy

Wild type and ΔΔ*ugd23* Arabidopsis plants were cultured *in vitro* and inoculated with J2 juveniles of *H. schachtii* as described above. Segments of roots containing syncytium were collected at 3, 5, and 10 days after inoculation. Control roots were collected from non-infected roots of two weeks old plants. Samples were immediately fixed in a mixture of 2% (w/v) paraformaldehyde (Sigma, St. Louis, Mi, USA) and 2% (v/v) glutaraldehyde (Fluka, Buchs, CH) in 0.05 M sodium cacodylate buffer (Sigma, St. Louis, Mi, USA) for 2 h at room temperature. Thereafter they were post-fixed in osmium tetroxide, dehydrated in ethanol and infiltrated with EPON resin (Fluka, Buchs, CH) [10], [28]. Ultrathin sections were taken on a Leica UCT ultramicrotome (Leica Microsystems, Wetzlar, D) and after staining with uranyl acetate and lead citrate examined in an FEI 268 D ‘Morgagni’ (FEI Comp., Hillsboro, Or, USA) transmission electron microscope equipped with an SIS ‘Morada’ (Olympus SIS, Münster, D) digital camera.

### Analysis of Cell Wall Composition

Arabidopsis plants were grown and infected with nematodes as described above. Two weeks after inoculation, root segments containing syncytia were cut out (nematodes were removed) and immediately frozen in liquid nitrogen (20–30 mg). Segments from uninfected plant roots were used as control. The material was homogenized and extracted twice in 70% ethanol, once in methanol-chloroform and finally extracted with acetone. The pellet was dried and hydrolysed in 200 µl 2 M TFA for 1 h at 120°C. TFA was removed in vacuum and the dry pellet containing the hydrolysed polymers was dissolved in 200 µl water. Samples were analysed by HPLC on a Dionex ICS 3000 system with a PA 20 column [24]. Identity of peaks was further verified by authenticity standards. There were at least 3 biological replicates carried out and data was analysed using single factor ANOVA (p<0.05). A Fisher LSD test was applied to further analyse the data.

### Immunostaining

Root sections were first blocked for 30 min in PBST-BSA (PBS +3% (w/v) BSA +0.05% (v/v) Tween-20). Primary antibodies were diluted in PBST and incubated with root sections for 2 h followed by five washes with PBST. Sections were further incubated for 2 h with a secondary antibody (Cy3 labelled goat anti rat; Dianova) diluted 1∶500 in PBST and were finally washed three times with PBS to remove unbound antibodies. Sections were mounted in Mowiol4.88 (Roth, Karlsruhe, Germany) and examined in a Leica TCS5 confocal laser microscope. Sections were excited at 561 nm and fluorescence emission was recorded between 570 and 620 nm. Images were processed and analyzed using the Leica TCS software.

## Supporting Information

Figure S1 **GUS analysis of syncytia associated with male nematodes at 10 dpi.** There was no expression for any of the *UGD* genes observed in male associated syncytia at 10 dpi. (A) *UGD1*, (B) *UGD2*, (C) *UGD3* and (D) *UGD4.*
(PDF)

Figure S2 **GUS analysis of uninfected roots.** Expression of GUS for *UGD1* (A, B), *UGD2* (C, D), *UGD3* (E) and *UGD4* (F, G) in uninfected control roots.
(PDF)

Figure S3 **AREX data base pictures and GeneCchip data.** Expression of *UGD1, UGD2, UGD3* and *UGD4* in roots of a 7 day old seedlings as extracted from the AREX database at http://bar.utoronto.ca/efp/cgi-bin/efpWeb.cgi?dataSource=Root *UGD1* (At1g26570), *UGD2* (At3g29360), *UGD3* (At5g15490) and *UGD4* (At5g39320).
(PDF)

Figure S4 **Comparison of anatomy and ultrastructure of wild-type and** ΔΔ***ugd23*** **roots.** Comparison of the anatomy and ultrastructure of uninfected wild type and double mutant plant roots at the early stage of secondary growth (A, B, F and G) and anatomy of syncytia (C–E and H–J) at three examined developmental time points: 3 dpi (C and H), 5 dpi (D and I) and 10 dpi (E and J). Light (A and F) and transmission electron microscopy figures (B–E and G–J) of wild type (A–E) and ΔΔ*ugd23* (F–J) roots. Scale bars 25 µm (A, C–F, and H–J) and 5 µm (B and G). N-nematode, S-syncytium, X-xylem.
(PDF)

Figure S5 **CLSM-immunostaining (JIM7, LM5, LM6).** Immunostaining of control root and 10 dpi syncytium by cell wall antibodies JIM7 (A–D), LM5 (E–H) and LM6 (I–L) for control root (A, B, E, F, I and J) and syncytium (C, D, G, H, K and L).
(PDF)

Table S1 ***UGD*** **GeneChip data**
(PDF)
