# Supplementary figures and images for: Cell Wall Ingrowths in Nematode Induced Syncytia Require UGD2 and UGD3
Source: PLoS One. 2012 Jul 26;7(7):e41515. doi: 10.1371/journal.pone.0041515 (PMC3406070; doi:10.1371/journal.pone.0041515)

A

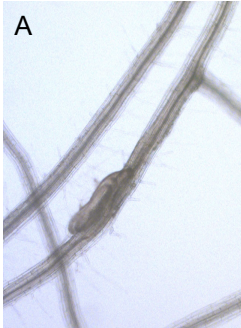

B

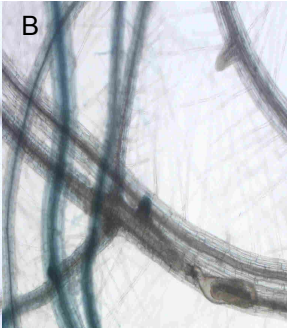

C

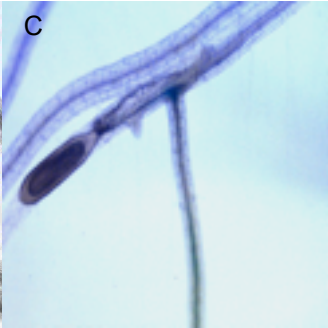

D

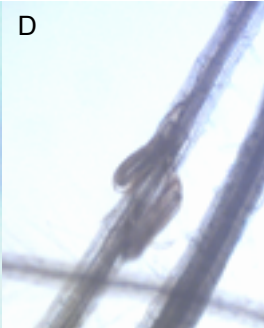

Supplement: Figure S1 — GUS analysis of syncytia associated with male nematodes at 10 dpi. There was no expression for any of the UGD genes observed in male associated syncytia at 10 dpi. (A) UGD1, (B) UGD2, (C) UGD3 and (D) UGD4. (PDF) [file pone.0041515.s001.pdf]

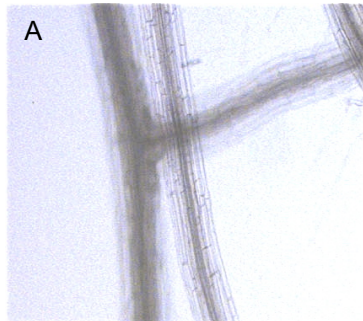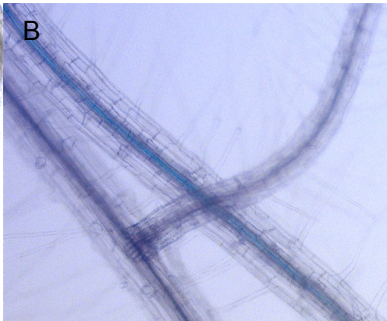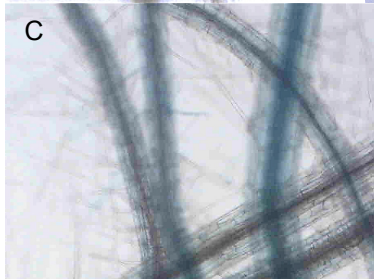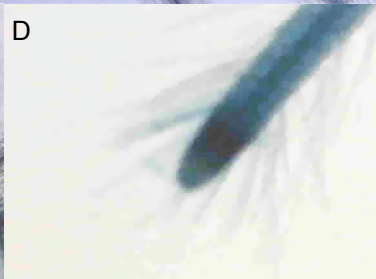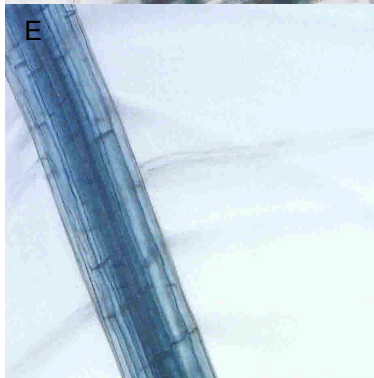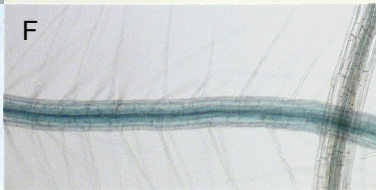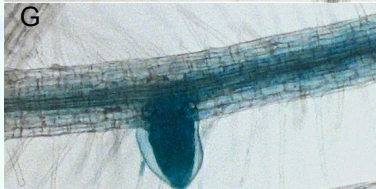

Supplement: Figure S2 — GUS analysis of uninfected roots. Expression of GUS for UGD1 (A, B), UGD2 (C, D), UGD3 (E) and UGD4 (F, G) in uninfected control roots. (PDF) [file pone.0041515.s002.pdf]

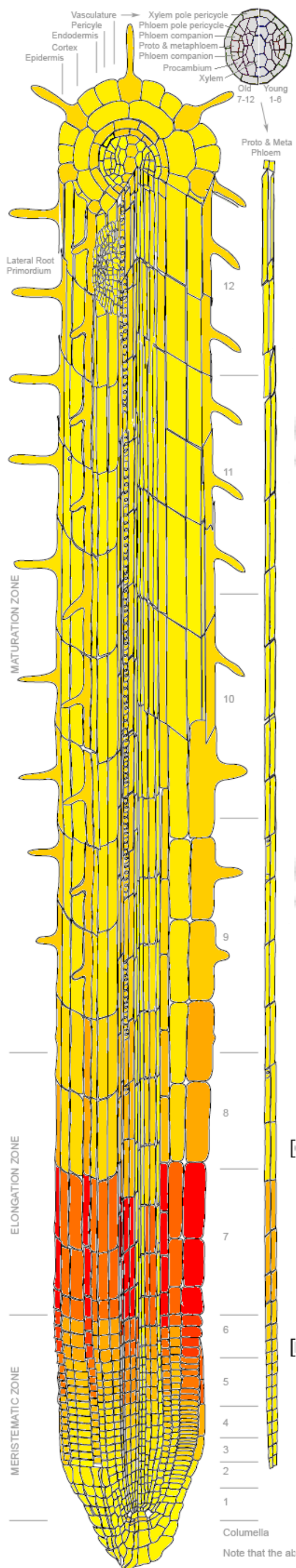

Supplement: Figure S3 — AREX data base pictures and GeneCchip data. Expression of UGD1, UGD2, UGD3 and UGD4 in roots of a 7 day old seedlings as extracted from the AREX database at http://bar.utoronto.ca/efp/cgi-bin/efpWeb.cgi?dataSource=Root UGD1 (At1g26570), UGD2 (At3g29360), UGD3 (At5g15490) and UGD4 (At5g39320). (PDF) [file pone.0041515.s003.pdf]

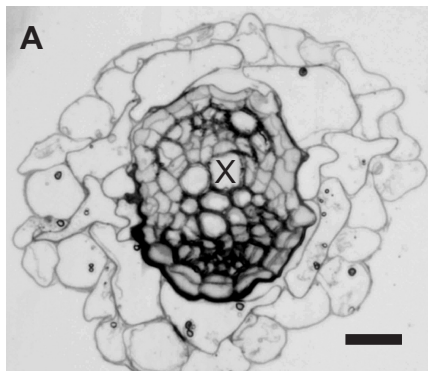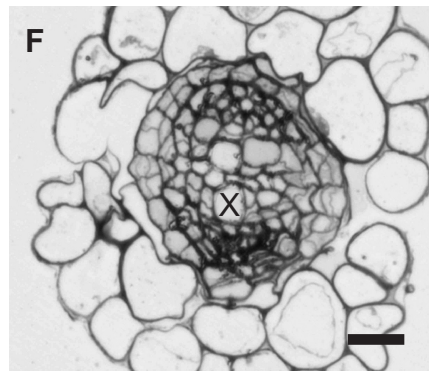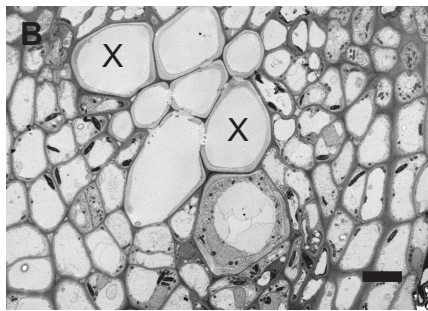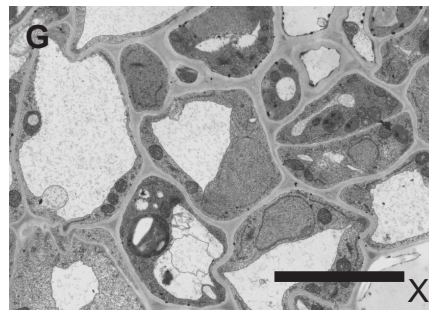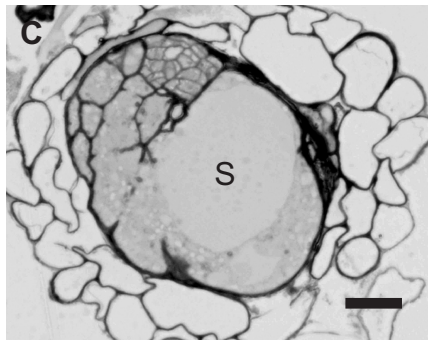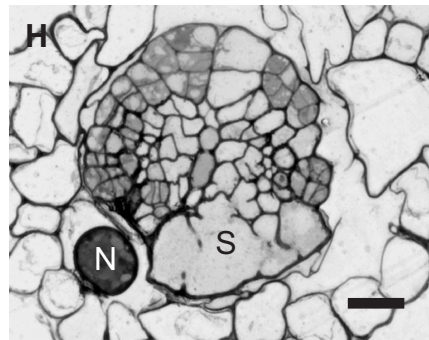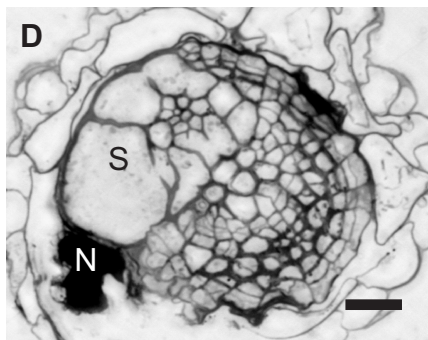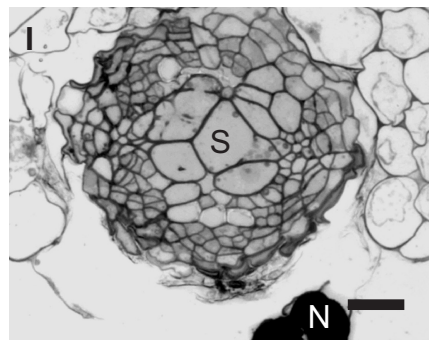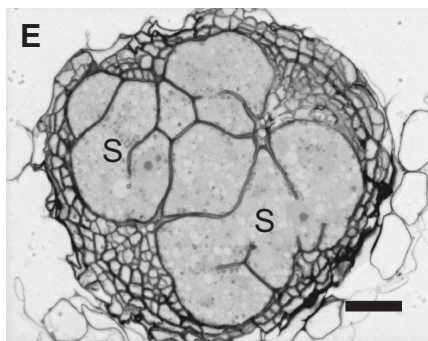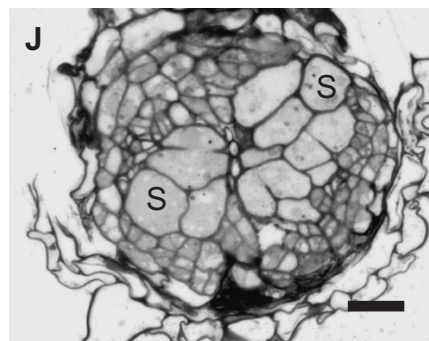

Supplement: Figure S4 — Comparison of anatomy and ultrastructure of wild-type and ΔΔugd23 roots. Comparison of the anatomy and ultrastructure of uninfected wild type and double mutant plant roots at the early stage of secondary growth (A, B, F and G) and anatomy of syncytia (C–E and H–J) at three examined developmental time points: 3 dpi (C and H), 5 dpi (D and I) and 10 dpi (E and J). Light (A and F) and transmission electron microscopy figures (B–E and G–J) of wild type (A–E) and ΔΔugd23 (F–J) roots. Scale bars 25 µm (A, C–F, and H–J) and 5 µm (B and G). N-nematode, S-syncytium, X-xylem. (PDF) [file pone.0041515.s004.pdf]

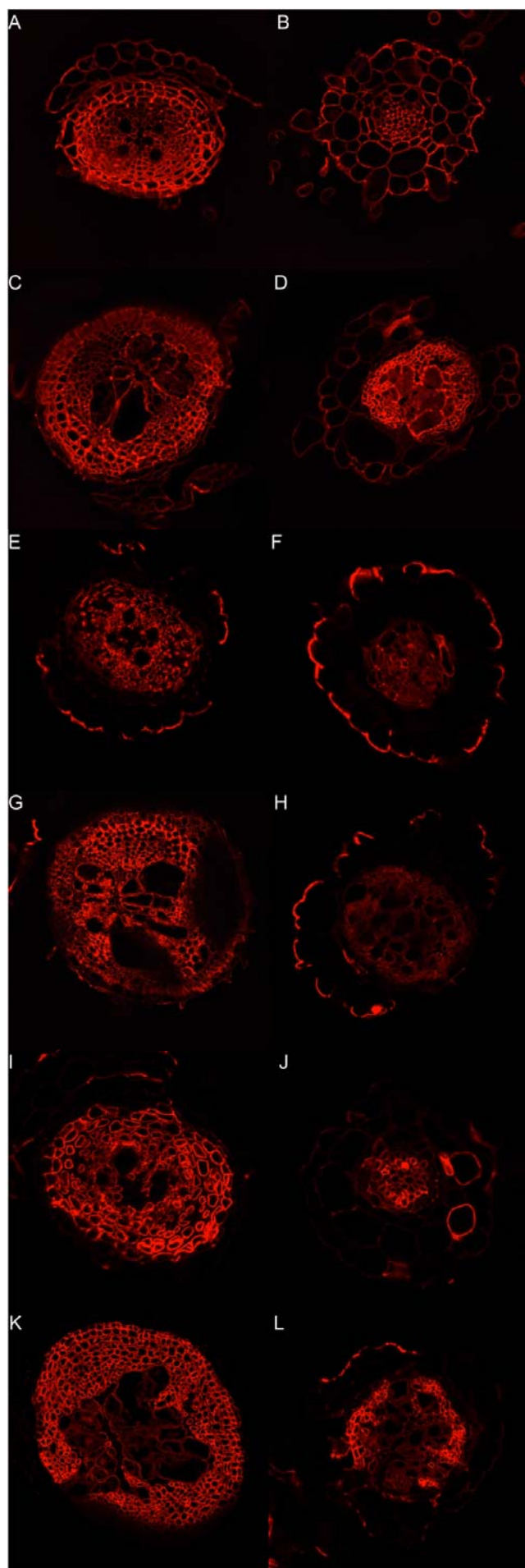

Supplement: Figure S5 — CLSM-immunostaining (JIM7, LM5, LM6). Immunostaining of control root and 10 dpi syncytium by cell wall antibodies JIM7 (A–D), LM5 (E–H) and LM6 (I–L) for control root (A, B, E, F, I and J) and syncytium (C, D, G, H, K and L). (PDF) [file pone.0041515.s005.pdf]
