# Supplementary material for: Cell Wall Ingrowths in Nematode Induced Syncytia Require UGD2 and UGD3
Source: PLoS One. 2012 Jul 26;7(7):e41515. doi: 10.1371/journal.pone.0041515 (PMC3406070; doi:10.1371/journal.pone.0041515)
Supplement: Table S1 — UGD GeneChip data (PDF) [file pone.0041515.s006.pdf]

Gene ID	*Gene*	Control	Syncytium (5 + 15 dpi)	Control vs syncytium	q-value	Enzyme function
At1g26570	*UGD1*	3.7	5.0	1.2*	0.01	UDP-glucose dehydrogenase
At3g29360	*UGD2*	5.7	5.3	-0.4	0.71	UDP-glucose dehydrogenase
At5g15490	*UGD3*	5.7	6.3	0.5	0.65	UDP-glucose dehydrogenase
At5g39320	*UGD4*	6.3	4.3	-2.0*	0.00	UDP-glucose dehydrogenase

Data from Szakasits et al. 2009 [14]
Data for microaspirated syncytia at 5 days post inoculation (5dpi) and 15 dpi were combined and compared with control roots. Elongation zone without root tip was used as control. All expression values have been normalized and are on a $\log_2$ scale (third and fourth column) and the differences (fold changes) between the pairwise samples displayed (fifth column) are accordingly normalized $\log_{2.}$
q-values indicate significance after correction for multiple testing controlling the false discovery rate.
* significant up-regulation or down-regulation
